# Machine learning-based prediction of postoperative nausea and vomiting after spinal anesthesia: A retrospective observational study

**DOI:** 10.1371/journal.pone.0333162

**Published:** 2026-08-05

**Authors:** Hiroshi Hoshijima, Tomo Miyazaki, Shinichiro Omachi, Daisuke Konno, Shigekazu Sugino, Masanori Yamauchi, Toshiya Shiga, Kentaro Mizuta

**Affiliations:** 1 Division of Dento-Oral Anesthesiology, Tohoku University Graduate School of Dentistry, Sendai, Miyagi, Japan; 2 Graduate School of Engineering, Tohoku University, Sendai, Miyagi, Japan; 3 Department of Anesthesiology and Perioperative Medicine, Tohoku University School of Medicine, Sendai, Miyagi, Japan; 4 Department of Anesthesiology and Pain Medicine, International University of Health and Welfare Ichikawa Hospital, Ichikawa, Chiba, Japan; University of the West of England, UNITED KINGDOM OF GREAT BRITAIN AND NORTHERN IRELAND

## Abstract

Postoperative nausea and vomiting (PONV) is a frequent and serious complication after surgery. PONV also reduces patient satisfaction with surgery under spinal anesthesia and increases medical costs due to prolonged hospitalization. The purpose of this study is to apply artificial intelligence (AI) machine learning analysis to identify risk factors for PONV in patients undergoing surgery with spinal anesthesia. This retrospective study used artificial intelligence to analyze data of adult patients (aged ≥20 years) who underwent surgery under spinal anesthesia at Tohoku University Hospital from January 1, 2010 to December 31, 2022. To evaluate PONV, patients who experienced nausea and/or vomiting or used antiemetics within 24 hours after surgery were extracted from postoperative medical records. The selected data were analyzed after propensity score matching with patients who did not experience PONV. We created an ensemble model for predicting the probability of PONV using five machine learning algorithms: random forest, gradient boosting machine, k-nearest neighbor, multilayer perceptron, and decision tree. Data were available for 4,574 patients. We performed propensity score matching and selected 538 patients for analysis (269 in the PONV group and 269 in the non-PONV group). The use of postoperative fentanyl was identified as the strongest contributor to PONV, followed by duration of surgery, body mass index (BMI), total urine output, and duration of anesthesia. The identified risk factors were female sex, BMI < 25 kg/m^2^, and duration of surgery (< 60 min), duration of anesthesia (< 100 min), cesarean section, use of postoperative fentanyl, administration of fentanyl/ morphine into the spinal arachnoid, and puncture level of epidural anesthesia (Th7–12) were identified as anesthesia/surgery-related risk factors. We used machine learning AI to evaluate risk factors for PONV after spinal anesthesia. We identified several patient-related and anesthesia/surgery-related risk factors for PONV. The AI analysis used in this study was accurate enough to identify risk factors for PONV during spinal anesthesia and may provide sufficient evidence for predicting PONV.

## Introduction

Postoperative nausea and vomiting (PONV) is a frequent and serious complication in patients undergoing surgery [1]. This complication decreases patient satisfaction with surgery and is associated with increased healthcare costs due to prolonged hospitalization. Numerous methods have been proposed to avoid PONV but have not completely prevented it [2].

The incidence of PONV ranges from 5% to 42% in patients undergoing lower extremity surgery, lower abdominal surgery, and cesarean section under spinal anesthesia [3–5]. Previous studies have identified several risk factors for PONV after spinal anesthesia compared with general anesthesia, including female sex, puncture level, and intraoperative hypotension. However, these factors remain controversial because they are very different from the known risk factors after general anesthesia.

Artificial intelligence (AI)-based technologies have evolved considerably and are starting to be applied in medicine [6,7]. The evolution of machine learning with AI, particularly deep learning methods such as neural networks and convolutional neural networks, is a driving force behind the development of AI. Deep learning differs from traditional machine learning in that AI learns differences between samples and selects the correct answer. Therefore, AI is uniquely capable of recognizing changes beyond human perception and establishing AI-specific identification methods.

The objective of the present study was to identify risk factors for PONV using machine learning analysis of AI in patients undergoing surgery with spinal anesthesia.

## Materials and methods

### Study setting and variables

This study was approved by the Ethics Committee of Tohoku University School of Medicine (#2023–31642, March 14, 2023). Before enrollment of any patients, the trial was registered in the UMIN Clinical Trials Registry (identifier UMIN000050012; principal investigator Hiroshi Hoshijima; registration date January 11, 2023). The study included patients aged older than 20 years who underwent surgery under spinal anesthesia at Tohoku University School of Medicine between 2010 and 2022. The date of access to the data for research purposes is April 19, 2023. The patients were divided into two groups according to whether they developed PONV. Exclusion criteria for this study age younger than 20 years, emergency surgery, surgery under general anesthesia, severe intraoperative or postoperative complications (cardiac arrest, severe arrhythmia, myocardial infarction, massive bleeding, asthma, pulmonary embolus), and admission to an intensive care unit after surgery.

Patient and surgical data were collected from the medical records, including the anesthetic record. We focused on data obtained within 24 h after surgery and collected the following information: sex, age, body mass index (BMI, kg/m^2^), duration of surgery, duration of anesthesia, puncture level for spinal anesthesia, puncture level for epidural anesthesia, type of local spinal anesthesia (isobaric or hyperbaric solution), drugs used during surgery (ephedrine [40 mg per ampoule], atropine [0.5 mg per ampoule], phenylephrine [1 mg per ampoule], nicardipine [2 mg per ampoule], hydrocortisone, prednisolone, oxytocin), administration of fentanyl/ morphine into the spinal arachnoid, postoperative use of fentanyl as epidural anesthesia, total infusion volume, total urine output, total blood loss, total blood transfusion, and intraoperative use of sedative agents (propofol, midazolam, dexmedetomidine) (Table 1). The type of surgery was classified as cesarean section or other surgery.

**Table 1:** Patients characteristics.

		Patients with PONV (269; 5.9%)	Patients without PONV (4,305; 94.1%)
Gender (Male/Female)		16 (5.9%)/253 (94.1%)	556 (12.9%)/3,749 (87.1%)
Age (year)		40.8 ± 15.2	39.5 ± 14.4
Body Mass Index (BMI, kg/mg-2)		25.2 ± 4.76	26.4 ± 19.8
Duration of anesthesia (min)		97.4 ± 45.5	61.2 ± 27.3
Duration of surgery (min)		68.1 ± 38.2	90.3 ± 33.1
Total infusion volume (ml)		1366.6 ± 622.6	1189.4 ± 585.4
Total bleeding loss (ml)		720.5 ± 735.2	613.8 ± 660.7
Total blood transfusion volume (ml)		38.9 ± 175.9	18.7 ± 131.7
Total urine output (n)		253.3 ± 250.7	201.6 ± 247.8
Caesarean section (n*)		73	1,175
Anesthesic agents			
	0.5%Bupivacaine (High specific gravity) (n*)	218	3,651
	0.5%Bupivacaine (Equal specific gravity) (n*)	51	654
	Fentanyl Intrathecal (n*)	82	1,704
	Fentanyl Post operative (0.25 mg/1A) (n*)		
	None	153	3127
	1 (A)	112	1399
	2 (A)	2	36
	3 (A)	0	4
	4 (A)	2	5
	5 (A)	0	2
	6 (A)	0	1
	Morphine (n*)	14	44
	Propofol (prefilled syringe) (mg**)	24	247
	Midazolam (n*)	5	105
	Dexmedetomidine (n*)	53	549
Puncture level of spinal anesthesia (n*)			
	L2-3	20	515
	L3-4	238	3554
	L4-5	9	226
	L5-S1	2	10
Puncture level of epidural anesthesia (n*)			
	None	47	982
	Th1–6	3	19
	Th7–12	182	2988
	L3	37	316
Cardiovascular agents			
	Atropine (n*)	5	132
	Ephedrine (n*)	77	1,479
	Nicardipine (n*)	4	36
	Phenylephrine (n*)	126	2,390
	Atonin(Oxxytocin) (n*)	192	3,077
Steroid			
	Hydrocortisone (n*)	0	17
	Prednisolone (n*)	1	12

* Total number of ampules or prefilled syringe used in each group.

**The amount of drug used (mg) is not the actual dose administered to the patients, but is calculated backwards from the number of ampules/vials of the drug administered to the patients.

The selected data will be subjected to propensity score matching to reduce bias due to confounding variables. Propensity score matching was employed to create a pair matched cohort with or without PONV. Propensity score was estimated using logistic regression in which all variables were input. Neighbor matching is used with cohort ratios of 1:1, 1:2, and 1:3, and is used for analysis in machine learning, with the result that yields the highest analytical accuracy being adopted. Performance of matching was evaluated using the absolute standardized mean difference (SMD) less than 0.1.

### Machine learning modeling

We constructed a model that predicts the probability of PONV and used it to determine the impact of items in the patient data. Next, we identified items that were risk factors for PONV according to their impact. Inspired by the works [8–10], we propose an ensemble model using five models constructed by algorithms: random forest, gradient boosting machine (GBM), k-nearest neighbor (KNN), multilayer perceptron (MLP), and decision tree algorithms [11]. The ensemble model uses each model to predict probability of PONV. Then, the final prediction is classified to PONV if the average of the probabilities is above threshold 0.5. We implemented the five models using the Scikit-learn [12]. We randomly divided the patient data into a training set (70%), a validation set (20%), and a test set (10%). We applied a hyper-parameter search to each model by 10-fold cross validation on the training dataset. Specifically, the training data was randomly split to ten subsets. We trained a model using nine subsets, the impact of the parameters was evaluated on the rest subset. We averaged the results of 10 subsets and selected the best parameters. Table 2 shows the hyper-parameters of the models.

**Table 2:** Hyper-parameters.

Model	Hyper-parameters
AdaBoost	Number of estimators = 200; Learning_rate = 0.8
Logistic	Inverse of regularization strength, C = 0.4; L2 norm penalty.
Forest	Estimators = 60; min_samples_leaf’ = 1; min_samples_split = 3
GBM	Max_depth = 10; n_estimators = 150; num_leaves = 31
KNN	Ball tree. algorithm; leaf size = 10; n_neighbors = 8
MLP	Tanh; batch size = 8; hidden_layer_sizes = 128; adam
SVC	Regularization C = 10.0; RBF kernel
Transformer	Number of head = 1; dim_feedforward = 1024; SGD, batch size = 16;
Tree	Max_features = 0.4; min_samples_leaf’ = 1; min_samples_split = 4;

We calculated the impact of each item using the Shapley value [13]. The Shapley value of an item represents its contribution to the prediction value. Equation (1) defines the Shapley value $\phi_{i}$ for item i in model f. R is the set of all items in the patient data, $P^{R}$ is the set of features including item i, and M is the number of items (*M* = 31 in this study). Thus, the Shapley value for item i is the expected value over sets of items with and without item i. We used an approximation framework to calculate the Shapley value [14].


$$\begin{array}{c} \begin{array}{c} \phi_{i}=\sum_{R\in \text{R}}\frac{\text{1}}{M!}\left[f\left(P^{R}\right)-f\left(P^{R}\setminus \{i\}\right)\right] \end{array} \end{array}$$ (1)

## Results

### Eligible data

We obtained data from the Tohoku University database on 5808 patients who underwent surgery under spinal anesthesia. Data for 1,234 patients were excluded (spinal puncture level unclear, n = 1,215; missing data, n = 12; no PONV adjudication, n = 3; no information on drugs used for spinal anesthesia, n = 3; concomitant general anesthesia, n = 3; and no surgery, n = 1), leaving data for 4,574 patients available for analysis. The patient characteristics are shown in Table 1. In total, 269 (5.9%) of the 4,574 patients developed PONV. As a result of propensity score matching, the analysis results for a cohort ratio of 1:1 were adopted, and 538 patients were selected (269 in the PONV group and 269 in the non-PONV group) (S1 Fig).

We evaluated the prediction models using the area under the curve (AUC) of the receiver-operating characteristic curves. In addition, we used three metrices to verify the classification performance. The precision, recall, and f-score are defined as:


$$\begin{array}{c} \text{precision}=\frac{TP}{TP+FP},\;\text{recall}=\frac{TP}{TP+FN},\;\text{f}-\text{score}=\frac{\text{2}TP}{\text{2}TP+FP+FN}. \end{array}$$

TP represents the number of true-positive samples, which are correctly classified to PONV by the models. Likewise, FP and FN are false-positive and false-negative, respectively.

We compared the proposed model with the seven algorithms: logistic regression, support vector classification (SVC), *k*-nearest neighbor (KNN), multi-layer perceptron (MLP), decision tree, adaboost, and gradient boosting machine (GBM). In addition, we constructed another comparison model based on transformer, which is the state-of-the-art architecture in machine learning. The transformer model is composed of two modules: the transformer encoder [15] and a fully connected layer, which are responsible for feature extraction and prediction, respectively.

S2 Fig shows the receiver-operating characteristic curves. Table 3 demonstrated the ability of our model to recognize PONV and warranting further analysis. Specifically, the AUC score was 0.81 for the ensemble model. The performance of the logistic model was insufficient. In contrast, the transformer model was sufficient. The ensemble model outperformed the baselines. Although the transformer model is the state-of-the-art for various tasks, the performance for PONV classification was moderate. This may be due to insufficient amount of the training data for the transformer model.

**Table 3:** Comparison of machine learning models.

Model	Accuracy	Recall	Precision	F value	AUC
Logistic	0.50	0.52	0.57	0.54	0.44
AdaBoost	0.63	0.63	0.65	0.67	0.59
SVC	0.61	0.65	0.67	0.66	0.61
Tree	0.72	0.81	0.74	0.77	0.73
MLP	0.70	0.77	0.73	0.75	0.73
Transformer	0.72	0.81	0.74	0.77	0.77
Forest	0.70	0.74	0.74	0.74	0.77
KNN	0.67	0.84	0.67	0.74	0.78
GBM	0.63	0.77	0.65	0.71	0.80
Ensemble	0.74	0.84	0.74	0.79	0.81

Next, we calculated the mean absolute Shapley Additive exPlanations (SHAP) value for each item by averaging the absolute SHAP values over the test data (S3 Fig). If an item had a SHAP value greater than 0, it was judged to be a risk factor for PONV.

### Risk factors for PONV

**Fig 1:**
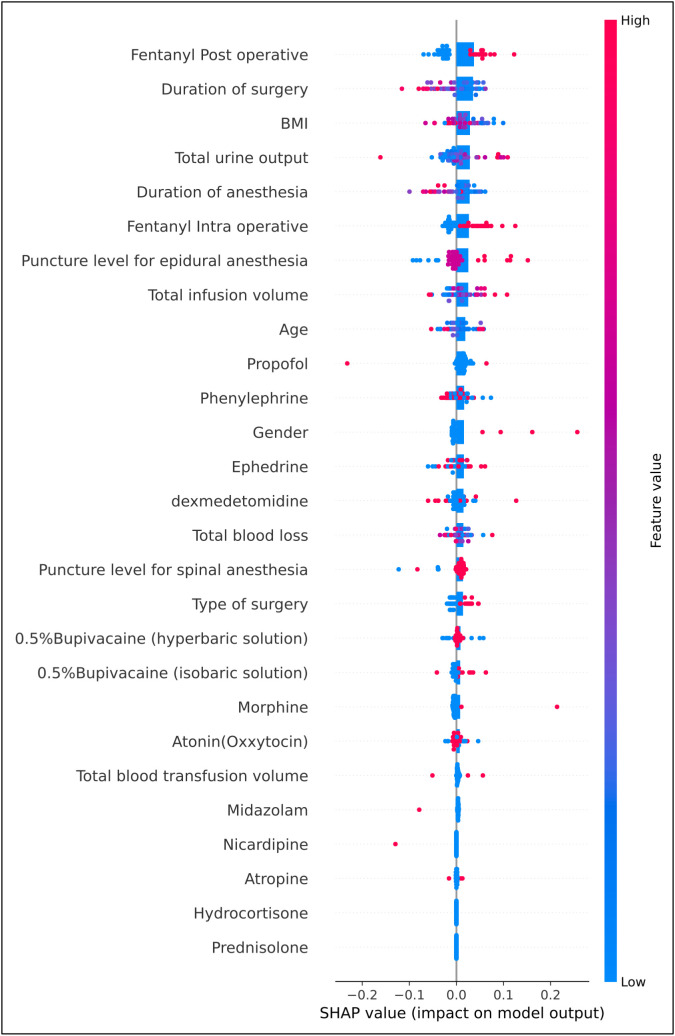
Summary of plot. The risk factors for PONV are plotted in descending order of relevance.

The use of postoperative fentanyl was the item most strongly associated with PONV after surgery under spinal anesthesia. Duration of surgery, BMI, total urine output, and duration of anesthesia were subsequently associated with PONV. (Fig 1).

**Fig 2:**
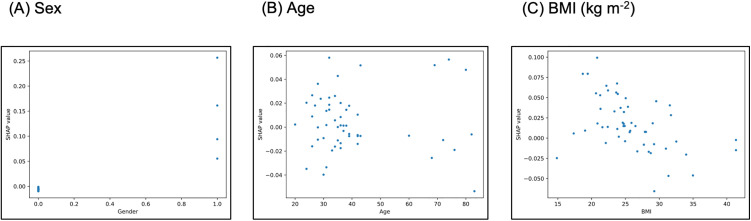
Relationship between patient-related and time-related risk factors and PONV in SHAP value. The X-axis represents (A) sex (0: male, 1: female), (B) age, and (C) BMI (kg m^2 −1^), The Y-axis represents the risk of PONV. SHAP value above 0 is related to the risk of PONV.

**Fig 3:**
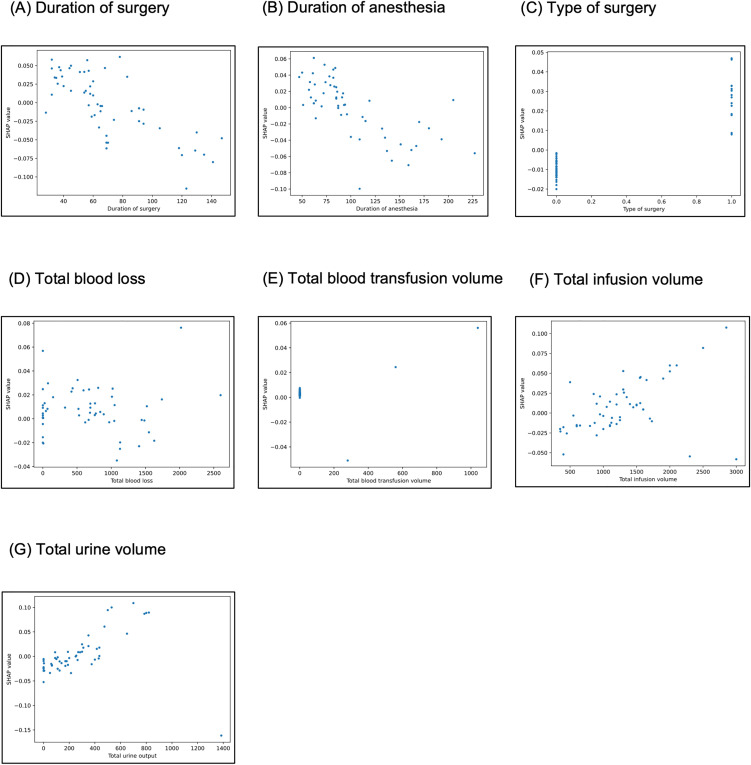
Relationship between fluid-related risk factors and PONV in SHAP value. The X-axis represents (A) duration of surgery (min), (B) duration of anesthesia (min), (C) Type of surgery (0:another surgery, 1: cesarean section), (D) total blood loss (ml), (E) total blood transfusion volume (ml), (F) total infusion volume (ml), and (G) total urine volume. The Y-axis represents the risk of PONV. SHAP value above 0 is related to the risk of PONV.

**Fig 4:**
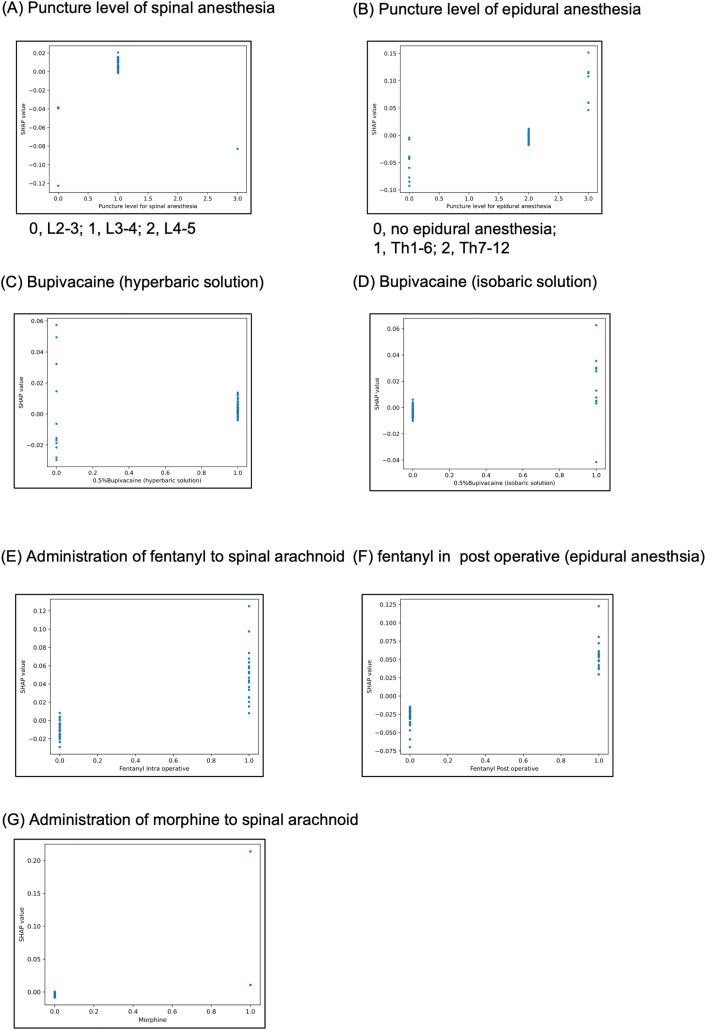
Relationship between anesthetic agent-related risk factors and PONV in SHAP value. The X-axis represents (A) Puncture level of spinal anesthesia (0: L 2-3, 1: L 3-4, and 2: L 4-5), (B) Puncture level of epidural anesthesia (0: No use of epidural, 1: Th1-6, and 2: Th 7-12), (C) Bupivacaine (hyperbaric solution), (D) Bupivacaine (isobaric solution), (E) Administration of fentanyl to spinal arachnoid, and (F) fentanyl in epidural anesthesia, and (G) Administration of morphine to spinal arachnoid. The Y-axis represents the risk of PONV. SHAP value above 0 is related to the risk of PONV.

**Fig 5:**
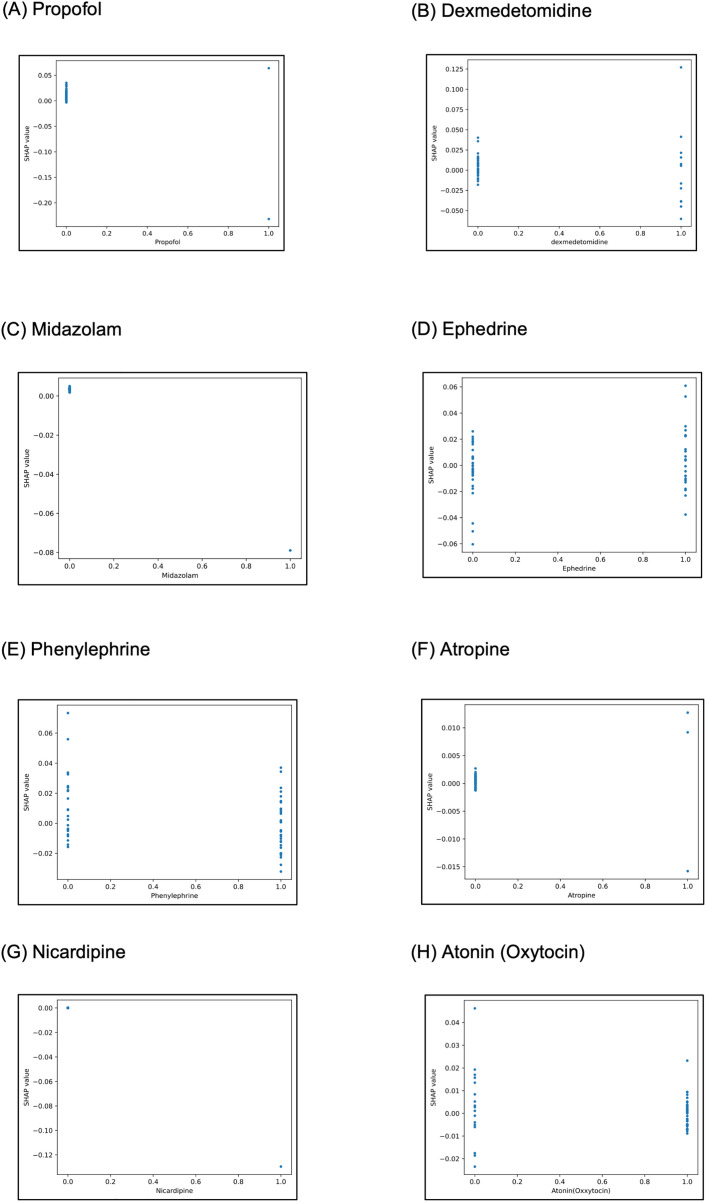
Relationship between anesthetic agent-related risk factors and PONV in SHAP value. The X-axis represents (A) Propofol, (B) Dexmedetomidine, (C) midazolam, (D) Ephedrine, (E) Phenylephrine, (F) Atropine, (G) Nicardipine, and (H) Atonin. The Y-axis represents the risk of PONV. SHAP value above 0 is related to the risk of PONV.

The analysis identified female sex as a patient-related risk factor for PONV (Fig 2A) and BMI (< 25 kg/m^2^) (Fig 2C). Anesthesia and surgical-related risk factors for PONV were postoperative fentanyl use as epidural anesthesia (Fig 4F), duration of surgery of less than 60 minutes (Fig 3A), duration of anesthesia (Fig 3B), type of surgery (Fig 3C), puncture level of epidural anesthesia (Fig 4B), and intrathecal use of fentanyl (Fig 4E)/ morphine (Fig 4G). The patient age (Fig 2B), total blood loss (Fig 3D), total blood transfusion (Fig 3E), total fluid infusion volume (Fig 3F), total urine output (Fig 3G), puncture level of spinal anesthesia (Fig 4A), use of bupivacaine (isobaric or hyperbaric solution) (Fig 4C and 4D), using propofol, dexmedetomidine, midazolam, ephedrine, phenylephrine, atropine, nicardipine, and oxytocin (Fig 5A–5H) did not have any clear relationship with PONV. The associations of hydrocortisone, and prednisolone, with PONV could not be analyzed because of the small number of samples.

## Discussion

In this study, use of postoperative fentanyl was the item most strongly associated with PONV after surgery under spinal anesthesia. Female sex and BMI (< 25 kg/m^2^) were identified as patient-related risk factors for PONV, and duration of surgery (< 60 min), duration of anesthesia (< 100 min), cesarean section, use of postoperative fentanyl, administration of fentanyl/ morphine into the spinal arachnoid, and puncture level of epidural anesthesia (Th7–12) were identified as anesthesia/surgery-related risk factors.

The central nervous system controls nausea and vomiting via two distinct components: the chemoreceptor trigger zone (CTZ) in the medulla and the vomiting center [16]. The CTZ, located in the area postrema at the base of the fourth ventricle, is a highly vascularized area where the blood-brain barrier is not well-developed. Meanwhile, the vomiting center is located in the lateral reticular formation of the medulla and is responsible for coordinating the emetic response. This center processes numerous excitatory inputs, including signals from the vagus sensory fibers of the gastrointestinal tract, the vestibular nuclei in the brainstem, higher cortical centers, the CTZ, and receptors sensitive to intracranial pressure. These areas are rich in dopamine, muscarinic, histamine, and opioid receptors [16]. In spinal anesthesia, PONV is thought to result from the inability to block vagal nerve activity during sympathetic blockade, combined with cerebral hypoxia due to reduced blood flow from a drop in blood pressure [5]. Nonetheless, multiple mechanisms likely contribute to PONV, and the specific role of each mechanism can vary and remains speculative in individual cases. Previous reports have identified factors associated with a higher likelihood of PONV after spinal anesthesia, including female sex, hypotension, level of block, baseline heart rate below 60 bpm, and history of motion sickness [17].

In this study, use of postoperative fentanyl was found to be the strongest risk factor for PONV. Fentanyl is well known to be a strong risk factor for PONV under spinal anesthesia [18]. Silvasti et al. previously reported that the use of fentanyl for patient-controlled anesthesia after surgery increased the incidence of PONV [19]. Chang et al. also reported that PONV increased when fentanyl was used for postoperative epidural anesthesia [18]. Opioids, including fentanyl, are known to be emetogenic in their own right, and mechanisms proposed for opioid-induced nausea and vomiting include direct stimulation of chemoreceptor sites, increased vestibular sensitivity, and delayed gastric emptying [20]. In this study, postoperative fentanyl use was found to be a risk factor for PONV, but it is also well known that postoperative pain increases the risk of PONV [21]. Furthermore, in this study, intrathecal administration of small amounts of fentanyl and morphine was also a risk factor for PONV. Previous reports have also shown that intrathecal morphine increases PONV. A 2020 report by Moraitis et al. investigated the incidence of PONV in patients undergoing hip and knee arthroplasty who underwent spinal anesthesia with bupivacaine 20 mg in combination with morphine 0.12 mg. Among these patients, 70% experienced PONV without antiemetic prophylaxis [22]. Similarly, a 2020 study by Selzer et al. also performed spinal anesthesia with 0.2 mg of intrathecal morphine for cesarean section. They reported that PONV occurred in 85% of these patients [23]. Thus, intrathecal morphine has been shown to increase PONV, even at very small doses.

Patient-related risk factors for PONV identified in this study were female sex and BMI (< 25). Female is a well-known risk factor for PONV [17], whereas the involvement of BMI in PONV remains controversial. Previous reports have shown that an increase in BMI does not affect PONV [24] and that low body weight does not increase the incidence of PONV [25]. On the other hand, there are also reports that the incidence of PONV increases with lower BMI [26]. Possible reasons for the increased PONV in patients with low BMI include the relatively higher amount of medication administered intrathecally, which promotes hypotension, and the possibility that the distribution volume of lipid-soluble narcotics decreases due to low BMI, increasing central stimulation and increasing PONV. However, recent reports suggest that BMI is not an independent factor in PONV in spinal anesthesia, but is strongly influenced by confounding factors such as age, gender, and surgical procedure (cesarean section) [27]. Many patients undergoing cesarean section are young women, and as a result, their BMI tends to be low. Therefore, it is possible that patients with a low BMI are naturally selected, and that the statistics are processed as if low BMI patients are more likely to develop PONV. In fact, in this study, gender and cesarean section were risk factors for PONV.

In this study, surgical duration of less than 60 minutes and anesthesia duration of less than 100 minutes were risk factors for PONV. It has been suggested that hemodynamic instability occurring immediately after spinal anesthesia is strongly associated with PONV. The peak of sympathetic blockade during early spinal anesthesia occurs within 10–20 minutes after intraspinal injection. Spinal anesthesia causes rapid sympathetic blockade, leading to hypotension, decreased venous return, and decreased cerebral perfusion. This hypotension during spinal anesthesia is said to be strongly associated with PONV [28,29].

### Limitations

The primary limitation of this study is the small sample size after propensity score matching. While this improved the accuracy of the machine learning method, it reduced the accuracy of the SHAP values. Additionally, insufficient sample size prevented adequate analysis of certain parameters. Another significant limitation is the lack of external validation. Generally, it is desirable to test the generality and predictive power of AI models on single-center datasets through external validation, but this was not performed in this study. A third limitation of this study is the bias in surgical techniques, including a high proportion of cesarean sections (27%, 1248/4574), which may have contributed to the study’s bias. Furthermore, some patients in this study received sedatives during surgery. While sedatives used during surgery may contribute to the risk of PONV, these factors could not be ruled out in the analysis.

## Conclusions

In this study, we used machine learning AI to analyze risk factors for PONV during spinal anesthesia and identified female gender, low BMI, intraoperative and postoperative fentanyl use, short surgery duration, short anesthesia duration, cesarean section, and non-use of midazolam. The AI analysis used in this study was accurate enough to identify risk factors for PONV during spinal anesthesia and may provide sufficient evidence for predicting PONV.

## Supporting information

S1 Fig Patient flow diagram. (DOCX)

S2 Fig ROC curves. Performance evaluation of the nine machine learning models, measured using a ROC curves. Support vector machine (SVC), Random Forest (Forest), k-nearest neighbor algorithm (KNN), Decision tree, Adaboost (ADA), Gradient boosting machine (GBM), Multi-Layer pPerceptron (MLP), Logistic, and Ensemble.
(DOCX)

S3 Fig Mean absolute SHAP value of each feature. (DOCX)
