# Supplementary material for: Machine learning-based prediction of postoperative nausea and vomiting after spinal anesthesia: A retrospective observational study
Source: PLoS One. 2026 Aug 5;21(8):e0333162. doi: 10.1371/journal.pone.0333162 (PMC13440857; doi:10.1371/journal.pone.0333162)
Supplement: Supplementary file 1 [file pone.0333162.s001.docx]

**Supporting information**

**Figure S1.** Patient flow diagram.
