# Supplementary material for: Machine learning-based prediction of postoperative nausea and vomiting after spinal anesthesia: A retrospective observational study
Source: PLoS One. 2026 Aug 5;21(8):e0333162. doi: 10.1371/journal.pone.0333162 (PMC13440857; doi:10.1371/journal.pone.0333162)
Supplement: Supplementary file 3 [file pone.0333162.s003.docx]

**Figure S3.** Mean absolute SHAP value of each feature.
